# Stefin B alleviates the gouty arthritis in mice by inducing the M2 polarization of macrophages

**DOI:** 10.1007/s00210-023-02911-w

**Published:** 2024-01-31

**Authors:** Shishui Lin, Xu Hu, Yang Li, Jiyue Huang, Rui Zhang, Xinxin Bai, Shaohuang Weng, Min Chen

**Affiliations:** 1 Department of Orthopedic Surgery, Fujian Medical University Union Hospital, No. 29 Xinquan Road, Gulou District, Fuzhou City, Fujian Province 350001 People’s Republic of China; 2 Department of Orthopedic Surgery, Shengli Clinical Medical College of Fujian Medical University, No. 134 East Street, Fuzhou City, Fujian Province 350001 People’s Republic of China; 3 Fujian Clinical Research Center for Spinal Nerve and Joint Diseases, No.134 East Street, Fuzhou City, Fujian Province 350001 People’s Republic of China; 4 Department of Orthopedics, 900TH Hospital of Joint Logistics Support Force, No. 156 West Second Ring North Road, Gulou District, Fuzhou City, Fujian Province 350025 People’s Republic of China; 5 Department of Pharmaceutical Analysis, School of Pharmacy, Fujian Medical University, No 1 North XueFu Road, Fuzhou City, Fujian Province 350122 People’s Republic of China

**Keywords:** Stefin B, Acute gouty arthritis, Macrophages polarization, IL-17

## Abstract

The present study aims to explore the therapeutic effect of Stefin B on gouty arthritis (GA) and the polarization of macrophages in mice. Stefin B-overexpressed or knockdown M0 macrophages were constructed. The GA model was established in mice by injecting 25 mg/mL MSU, followed by a single injecting of Stefin B-overexpressing adenovirus vector (GA model + Stefin B OE) or an empty vector (GA model + Stefin B OE NC). Stefin B was found lowly expressed in M1 macrophages. CD206 was markedly upregulated and IL-10 release was signally increased in Stefin B-overexpressed macrophages. In gouty arthritis mice, marked redness and swelling were observed in the ankle joint. Dramatical infiltration of inflammatory cells was observed in the GA model and GA model + Stefin B OE NC groups, which was suppressed in the Stefin B OE group. Increased proportion of F4/80^+^CD86^+^ cells observed in GA mice was markedly repressed by Stefin B overexpression, accompanied by the declined level of Caspase-1 and IL-17. Collectively, Stefin B alleviated the GA in mice by inducing the M2 polarization of macrophages.

**Supplementary Information:**

The online version contains supplementary material available at 10.1007/s00210-023-02911-w.

## Introduction

Acute gouty arthritis (GA) is a common arthropathy triggered by the accumulated crystals. As the precipitation of monosodium urate (MSU) crystals in the joint capsule, synovium, and bone tissues, the inflammation responses will be triggered to induce damage to bone or cartilage, accompanied with pain and restricted range of motion, which finally contribute to the development of GA (Ankli and Krahenbuhl 2016). Due to the impact of eating habits like excessive consumption of purine-rich foods or drinks and environmental factors, an increasing morbidity worldwide and in China was observed (Koto et al. 2021; Liu et al. 2021). Moreover, several complications, such as hypertension and cardiovascular disease, are reported on GA, which evoked challenges for the treatment of GA (Giannopoulos et al. 2019). Currently, medicines for GA mainly include nonsteroidal anti-inflammatory drugs (NSAIDs) and hypouricemic drugs, which are highly associated with severe gastrointestinal or cardiovascular adverse effects (Fields 2019). Therefore, it is urgent to develop safe and effective anti-GA agents.

Macrophages are derived from monocytes and can be polarized into M1 and M2 macrophages under different situations. M1 macrophages are mainly functioned as inflammatory cells for the elimination of tumor cells or microorganisms, while M2 macrophages are mainly functioned as anti-inflammatory cells to stimulate the growth of tumor (Ren et al. 2019). It is reported that the switch of M1/M2 macrophages is involved in GA development (Wang 2021). However, the potential mechanism underlying the function of M1/M2 macrophages in GA remains unclear. In gouty arthritis, MSU crystal deposition in the joints and surrounding tissues leads to acute inflammation, during which macrophages tend to express the pro-inflammatory phenotype CD86 of M1 type (So et al. 2018). During the spontaneous remission of gouty arthritis, macrophages produce TGF-β after being alerted by irritants, reducing the secretion of pro-inflammatory cytokine IL-1β and increasing the expression level of anti-inflammatory factor IL-10, polarizing M0 to M2 type, limiting inflammation development, and inducing inflammation resolution (Yang et al. 2021). Therefore, it is necessary to find effective mechanisms to regulate the polarization of macrophages from M0 to M2 type.

Stefin B, an endogenous cysteine cathepsin inhibitor localized in the cytosol, nucleus, and mitochondria, has been reported to be essential in the immune responses (Maher et al. 2014a). A previous study showed that Stefin B-knockout mice were more sensitive to lipopolysaccharide (LPS)-induced sepsis, accompanied by an increased secretion of pro-inflammatory cytokines in serum and an activated NLRP3 inflammasome (Trstenjak Prebanda et al. 2019). Moreover, a recent study has found that RfCytB, an inhibitor of Stefin B, is highly expressed in LPS-stimulated mouse macrophages, which further induces the expression of pro-inflammatory disease-related genes, such as iNOS and TNF-α (Wickramasinghe et al. 2020). In the present study, the possible efficacy of macrophage polarization regulated by Stefin B in GA mice was investigated to point out the potential value of Stefin B as the drug target for GA.

## Materials and methods

### Macrophages, polarization stimulation, and treatments

THP-1 cells were purchased from BeNa Culture Collection (BNCC288554, China). M0 macrophages were generated by stimulating THP-1 cells with 50 nM PMA for 24 h, which were stimulated by 100 ng/mL LPS and 20 ng/mL IFN-γ for 48 h to obtain M1 macrophages and stimulated by 20 ng/mL il-4 for 48 h to obtain M2 macrophages, respectively. To construct Stefin B-overexpressed M0 macrophages, THP-1 cells were stimulated by 50 nM PMA for 24 h, followed by transfecting the adenovirus containing the Stefin B-overexpressed vector (pcDNA3.1-Stefin B) for 48 h. The empty vector (pcDNA3.1-NC) was taken as a negative control. To construct Stefin B-knockdown M0 macrophages, THP-1 cells were stimulated by 50 nM PMA for 24 h, followed by transfecting siRNAs (Si-Stefin B-1, Si-Stefin B-2, and Si-Stefin B-3) targeting Stefin B for 48 h, respectively. The transfection efficacy was identified by the RT-PCR and western blotting assay. The sequences of siRNAs are listed in Table 1.

**Table 1:** Sequences of siRNAs

SiRNAs	siRNA sequences (5′-3′)
Si-Stefin B-1 F	GGACAAACUACUUCAUCAATT
Si-Stefin B-1 R	UUGAUGAAGUAGUUUGUCCTT
Si-Stefin B-2 F	CCAACAAAGCCAAGCAUGATT
Si-Stefin B-2 R	UCAUGCUUGGCUUUGUUGGTT
Si-Stefin B-3 F	GGUCCCAGCUUGAAGAGAATT
Si-Stefin B-3 R	UUCUCUUCAAGCUGGGACCTT
Si-NC F	UUCUCCGAACGUGUCACGUTT
Si-NC R	ACGUGACACGUUCGGAGAATT

### RT-PCR assay

Total mRNA was extracted from macrophages or the ankle joint tissue according to Trizon reagent instructions (CW0580S, CWBIO, China). cDNA synthesis was performed by RT-PCR reverse transcription kit operation. The HiScript II Q RT SuperMix for qPCR (+ gDNA wiper) (R223-01, Vazyme, China) was utilized to conduct the PCR reaction with the 2 × SYBR Green PCR Master Mix kit (A4004M, Lifeint, China). The internal reference gene was β-actin and the gene level was determined utilizing the 2^−ΔΔCt^ method. The sequences of primers are shown in Table 2.

**Table 2:** Primers of sequences

Genes	Sequences (5′-3′)
β-actin F	TGGCACCCAGCACAATGAA
β-actin R	CTAAGTCATAGTCCGCCTAGAAGCA
TNF-α F	CGAGTGACAAGCCTGTAGCC
TNF-α R	TGAAGAGGACCTGGGAGTAGAT
Caspase-1 F	AATACAACCACTCGTACACGTC
Caspase-1 R	AGCTCCAACCCTCGGAGAAA
CD206 F	CTCTACAAGGGATCGGGTTT
CD206 R	TGGTCAGCGGGTCTTTATT
Stefin B F	GCCGTGTCATTCAAGAGCC
Stefin B R	TCGCAGGTGTACGAAGTCC

### Western blotting assay

Macrophages were collected for the extraction of total proteins, which were quantified with the BCA method (E-BC-K318-M, Elabscience, USA), followed by conducting the separation with the 12% SDS-PAGE. After transferring proteins to the PVDF membrane, 5% skim milk was applied for blocking. The primary antibodies against Stefin B (14,754–1-AP, 1:500, Proteintech, USA) and GAPDH (TA-08, 1:2000, ZSGB-Bio, China) were added, followed by incubation with the secondary antibody (ZB-2301, 1:2000, ZSGB-Bio, China). After 60 min incubation, the ECL solution was added for exposure, and the expression level was quantified with the ImageJ software.

### Flow cytometry

A total of 10^6^ cells were used as negative control. Cells were collected and centrifuged to remove the supernatant, followed by suspended with PBS and centrifuged at 1500 rpm for 5 min. A total of 5 µL of antibodies for CD86 APC (374,207, Biolegend, China) and CD206 FITC (321,103, Biolegend, China) were added. After slight mixing, cells were incubated in the dark at room temperature for 20 min, followed by adding 1 mL PBS and centrifugated at 1500 rpm for 5 min. Cells were resuspended using 1 mL PBS, which were loaded onto the flow cytometry (NovoCyte 2060R, ACEA, China).

### ELISA assay for cytokine detection

ELISA kits were utilized for the detection of IL-6 (EK206/3–96, MULTI SCIENCE, China), TNF-α (EK282/4–96, MULTI SCIENCE, China), and IL-1β (E-EL-H0149c, Elabscience, China) released by macrophages, as well as IL-17 (MM-0170M1, MEIMIAN, China) in the serum of mice. The supernatant of macrophages or serum of mice was collected and diluted at a 1:1 ratio. The samples were loaded into wells, followed by adding 50 µL biotin-labeled antibody and culturing for 1 h at 37 °C. A total of 80 µL HRP-loaded secondary antibody was introduced following clearing the solution. After 10 min incubation at 37 °C, 50 µL TMB substrates were introduced and cultured at 37 °C for 10 min. Following loading 50 µL stop solution and detecting the OD value utilizing a microplate reader (WD-2102B, LIUYI, China).

### The construction of GA model in mice

A total of 24 C57BL/6 male, SPF grade mice were obtained from Suzhou Xishan Biotechnology Co., Ltd. To construct the GA model, each mouse was injected with 28 µL MSU crystals into the ankle joint cavity at a concentration of 25 mg/mL. After mice were anesthetized, the ankle circumference of the mice was measured three times by the suture method, and the average value was used as the ankle circumference before modeling. After measurement, the tibia was disinfected with iodophor, and the foot was bent at a 45° angle. A syringe was inserted into the ankle joint cavity from the lateral side of the right ankle joint at a 45° angle to the tibia. After the injection, significant redness and swelling were observed in the joint, and the circumference of the ankle joint was measured three times by the suture method. The average value was taken as the circumference of the ankle joint after modeling. Animals were divided into 4 groups, Control, GA model, GA model + Stefin B OE NC, and GA model + Stefin B OE, with 6 mice in each group. Mice in the GA model + Stefin B OE NC and GA model + Stefin B OE were injected with a single dose of adenovirus containing pcDNA3.1-Stefin B and pcDNA3.1-NC on day 1 and 4, respectively. The state of joint swelling was observed before modeling and 1 week after modeling. Changes in hair color, appetite, mental state, joint color, and swelling were observed during the animal experiment. One week after modeling, mice in each group were sacrificed, and ankle joint tissues and serum were collected.

### HE staining

The ankle joint tissues were rinsed with running water for several hours, followed by being dehydrated, embedded in paraffin, and sectioned. Samples were baked, then dewaxed, hydrated, and treated with hematoxylin and eosin reagent. Finally, samples were examined under a microscope (Olympus, Japan).

### Double immunofluorescence staining

Paraffin sections were baked, hydrated, and placed in a repair box for repair by adding pepsin, and permeabilized with 0.5% Triton X-100 at room temperature. Sections were blocked with 5% BSA for 30 min and then incubated with primary antibodies, including CD86 (1:100, DF6332, Affinity, USA) or CD206 (1:100, DF4149, Affinity, USA) in a wet box at 4 °C overnight. After being rinsed with PBS, the diluted fluorescent blue secondary antibody (1:100, As007, ABclonal, China) was added to the sections. After being rinsed with PBS, 5% BSA was dropped for blocking. A sufficient amount of diluted primary antibody, including F4/80 (1:100, DF2789, Affinity, USA), was dropped on sections that were incubated in a wet box. After being rinsed with PBS, the diluted fluorescent secondary antibody CY3 (1:100, As007, ABclonal, China) was dropped onto sections that were incubated in a wet box. The sections were mounted with an antifade mounting medium and observed under the fluorescence microscope (CKX53, Olympus, Japan).

### Statistical analysis

SPSS 20.0 software was used for statistical analysis. The quantitative data were expressed as mean ± standard deviation (SD). One-way ANOVA was performed for analysis, and the LSD method was used for pairwise comparison. *P* < 0.05 was taken as a significant difference.

## Results

### The identification of macrophage polarization from THP-1 cells

**Fig. 1:**
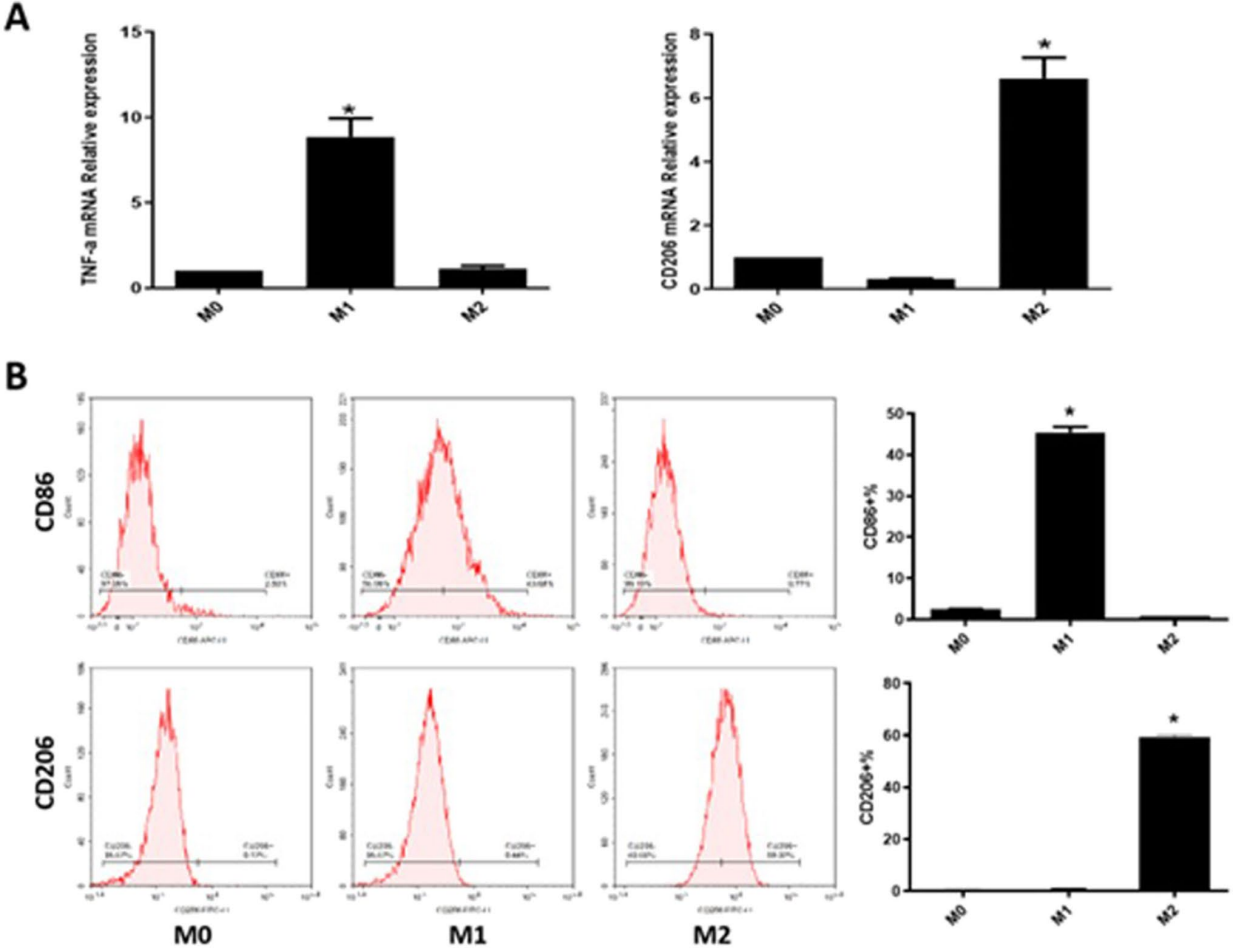
Macrophage polarization from THP-1 cells was identified. **A** The mRNA level of TNF-α was detected by the RT-PCR assay. **B** The level of CD86 and CD206 was checked by flow cytometry (*p < 0.05 vs M0)

As shown in Fig. 1A, compared to M0 macrophages, dramatically increased TNF-α level was observed in LPS- and IFN-γ-stimulated M0 macrophages, which was not changed in IL-4-stimulated M0 macrophages. Furthermore, compared to M0 macrophages, markedly elevated CD206 level was observed in IL-4 stimulated M0 macrophages, which was rarely changed in LPS and IFN-γ stimulated M0 macrophages. As shown in Fig. 1B, the percentage of CD86^+^ cells in LPS- and IFN-γ-stimulated M0 macrophages were approximately 50%, which was as low as 0.77% in IL-4-stimulated M0 macrophages. Moreover, the percentage of CD206^+^ cells in LPS- and IFN-γ-stimulated M0 macrophages were approximately 0.44%, which was as high as 60% in IL-4-stimulated M0 macrophages. These results confirmed the successful induction of M1 and M2 macrophages.

### Stefin B was differentially expressed in M1/M2 macrophages

**Fig. 2:**
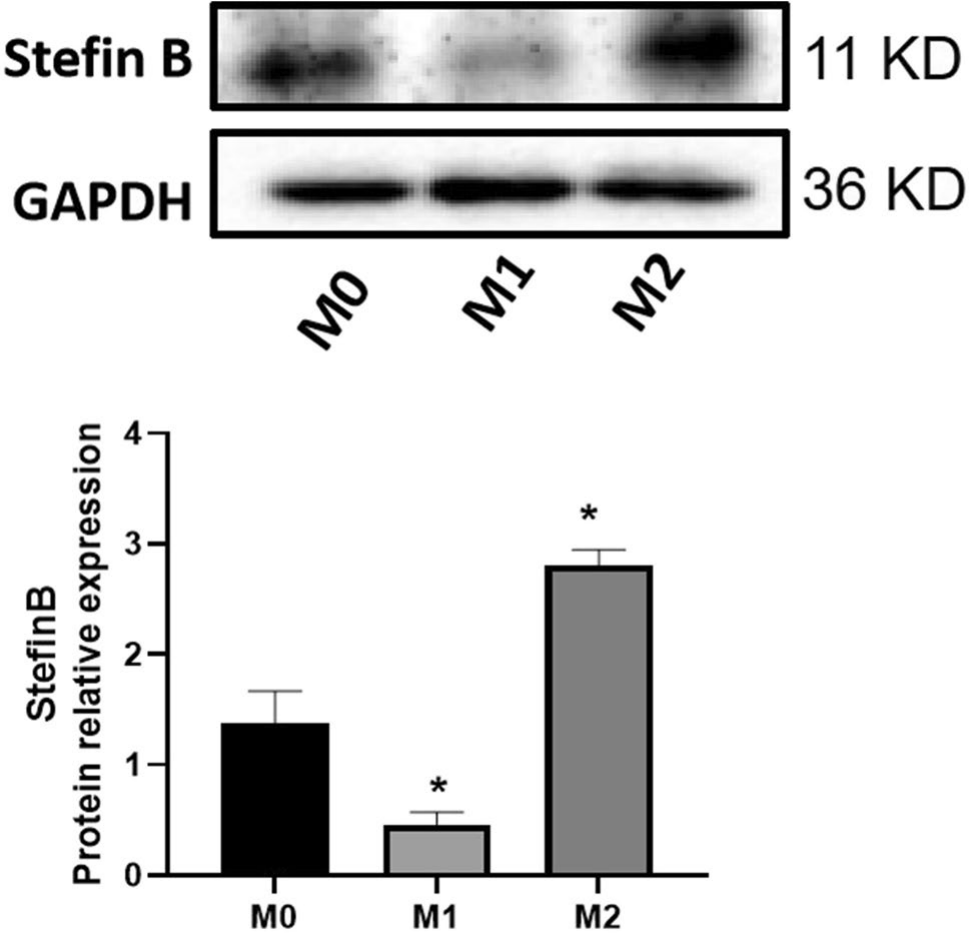
Stefin B was differentially expressed in M1/M2 macrophages. The protein level of Stefin B in M0, M1, and M2 macrophages was determined by western blotting assay (*p < 0.05 vs M0)

The protein level was checked following stimulating M0 macrophages with LPS and IFN-γ or IL-4. As shown in Fig. 2, compared to M0 macrophages, Stefin B level was markedly repressed in M1 macrophages and was dramatically elevated in M2 macrophages, suggesting the expression of Stefin B in macrophages might be facilitated by M2 polarization and repressed by M1 polarization.

### The identification of the overexpression and knockout of Stefin B in M0 macrophages

**Fig. 3:**
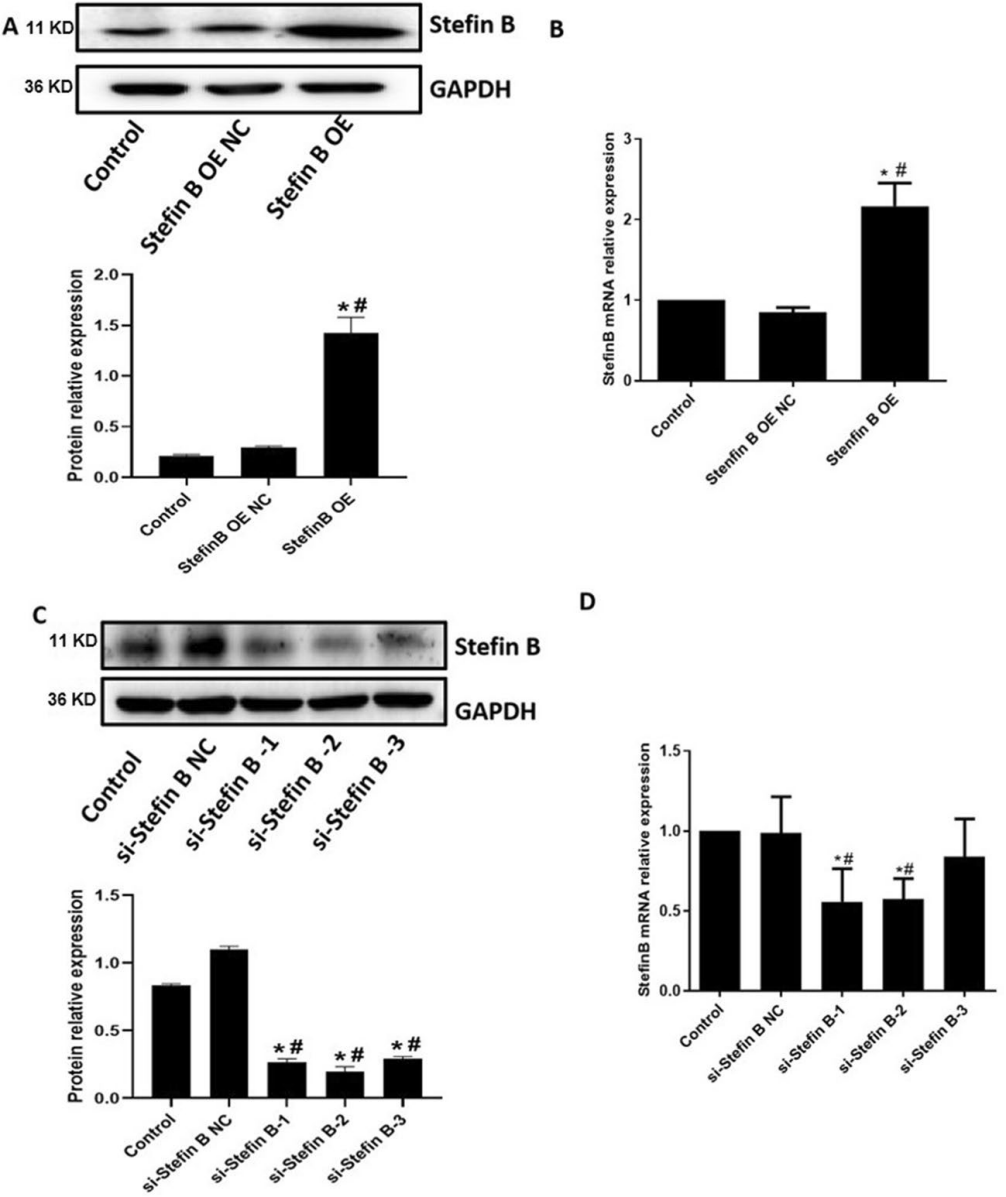
The identification of the overexpression and knockout of Stefin B in M0 macrophages. The level of Stefin B in pcDNA3.1-Stefin B-transfected M0 macrophages was checked by RT-PCR (**A**) and western blotting assay (**B**). The Stefin B expression in siRNAs-transfected M0 macrophages was checked by RT-PCR (**C**) and western blotting assay (**D**). (*p < 0.05 vs Control, #*p* < 0.05 vs Stefin B OE NC or si-Stefin B NC)

As shown in Fig. 3A, B, compared to the control and Stefin B OE NC group, Stefin B was found significantly upregulated in the Stefin B OE group. Furthermore, compared to the control and si-Stefin B NC group, Stefin B mRNA level was found greatly repressed in the si-Stefin B-1 and si-Stefin B-3 groups, while the protein level was signally repressed the si-Stefin B-1, si-Stefin B-2, and si-Stefin B-3 groups (Fig. 3C, D). Collectively, si-Stefin B-1 was chosen for the knockdown of Stefin B in M0 macrophages.

### The impact of Stefin B on the polarization of macrophages

**Fig. 4:**
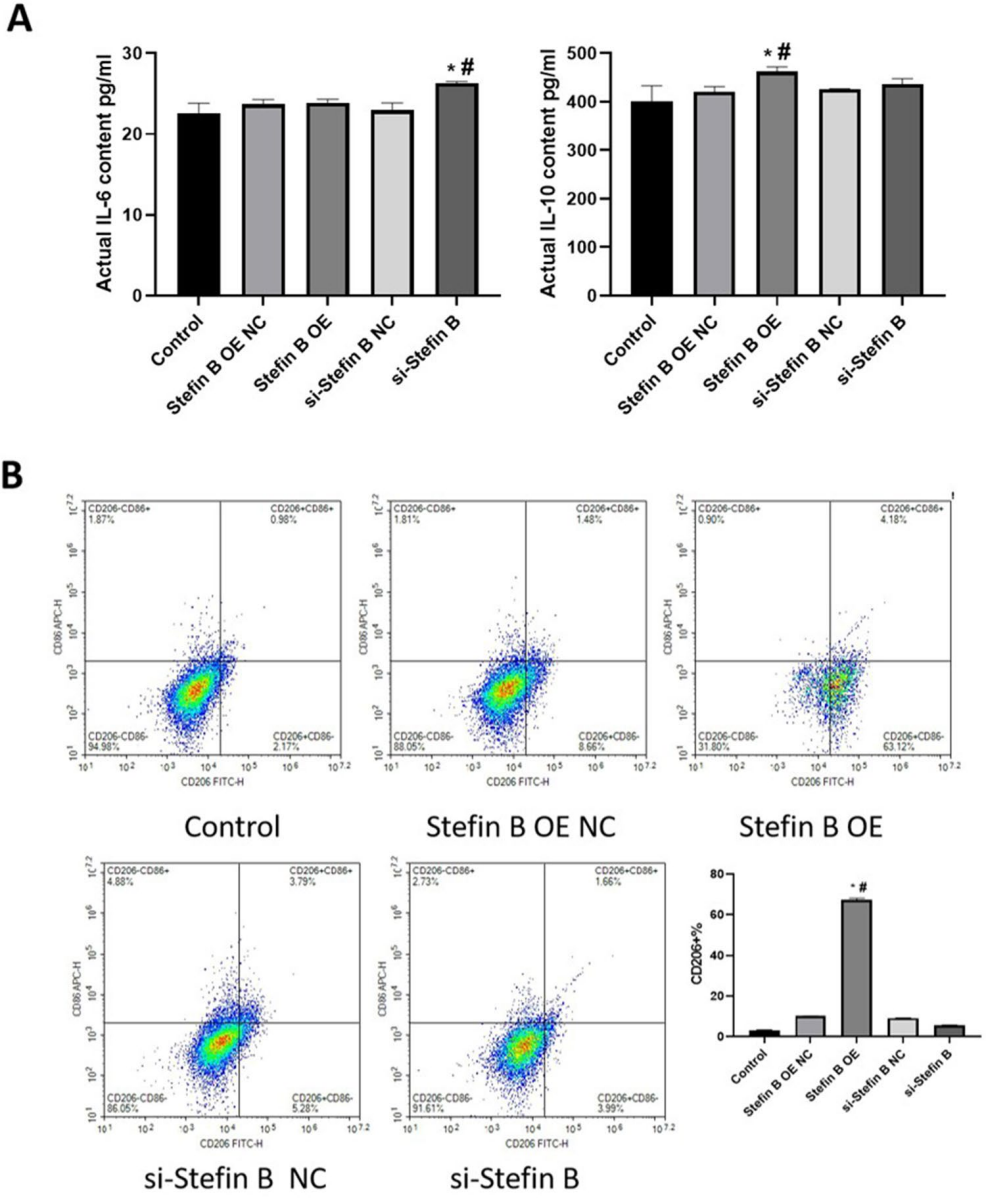
The M2 polarization could be facilitated by Stefin B. **A** The level of IL-6 and IL-10 released by macrophages was checked by ELISA. **B** The percentage of CD206 + macrophages was determined by the flow cytometry assay (*p < 0.05 vs Control, #*p* < 0.05 vs Stefin B OE NC or si-Stefin B NC)

The level of IL-6 and IL-10 was checked to determine the impact of Stefin B on the polarization of macrophages. As shown in Fig. 4A, compared to control and si-Stefin B NC, IL-6 level was significantly increased in the si-Stefin B group. Compared to control and Stefin B OE NC, IL-10 level was significantly elevated in the Stefin B OE group. These data suggested that the inflammation in macrophages could be repressed by Stefin B. Furthermore, compared to control and Stefin B OE NC, CD206 content was markedly increased to 60% in the Stefin B OE group. These results suggested that the M2 polarization could be facilitated by Stefin B.

### The identification of Stefin B-overexpressed adenovirus vector

**Fig. 5:**
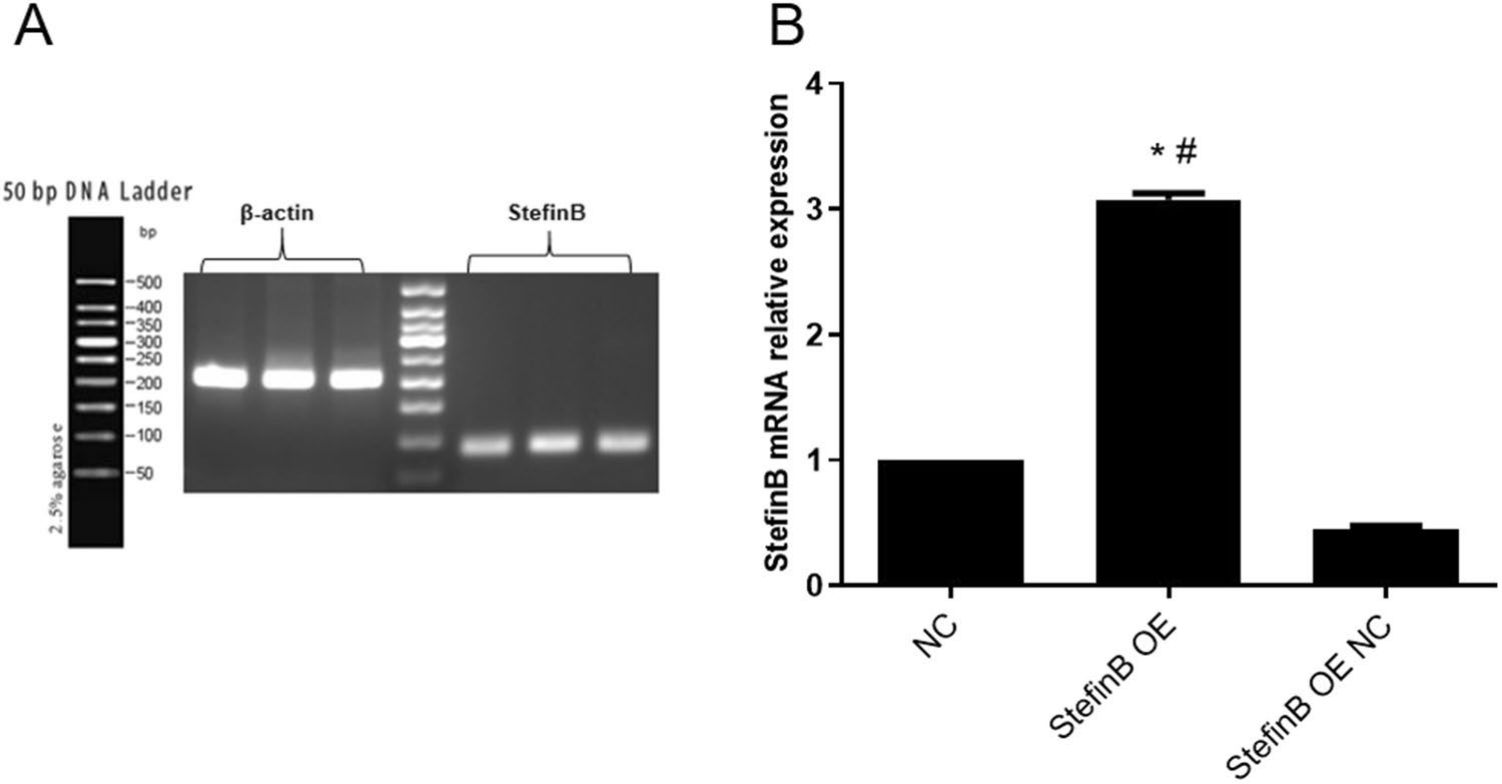
The identification of Stefin B-overexpressed adenovirus vector. **A** The picture of the gel electrophoresis. **B** The mRNA level of Stefin B was checked by RT-PCR (*p < 0.05 vs Control, #*p* < 0.05 vs Stefin B OE NC)

Stefin B-overexpressed adenovirus vector was constructed and packaged. DNA electrophoresis (Fig. 5A) showed bands of β-actin and Stefin B with normal brightness and correct position, which were target products. Compared to control and Stefin B OE NC group, Stefin B was found signally upregulated in the Stefin B OE group (Fig. 5B). These data indicated that the Stefin B-overexpressed adenovirus vector was successfully constructed.

### The successful establishment of GA model in mice

**Fig. 6:**
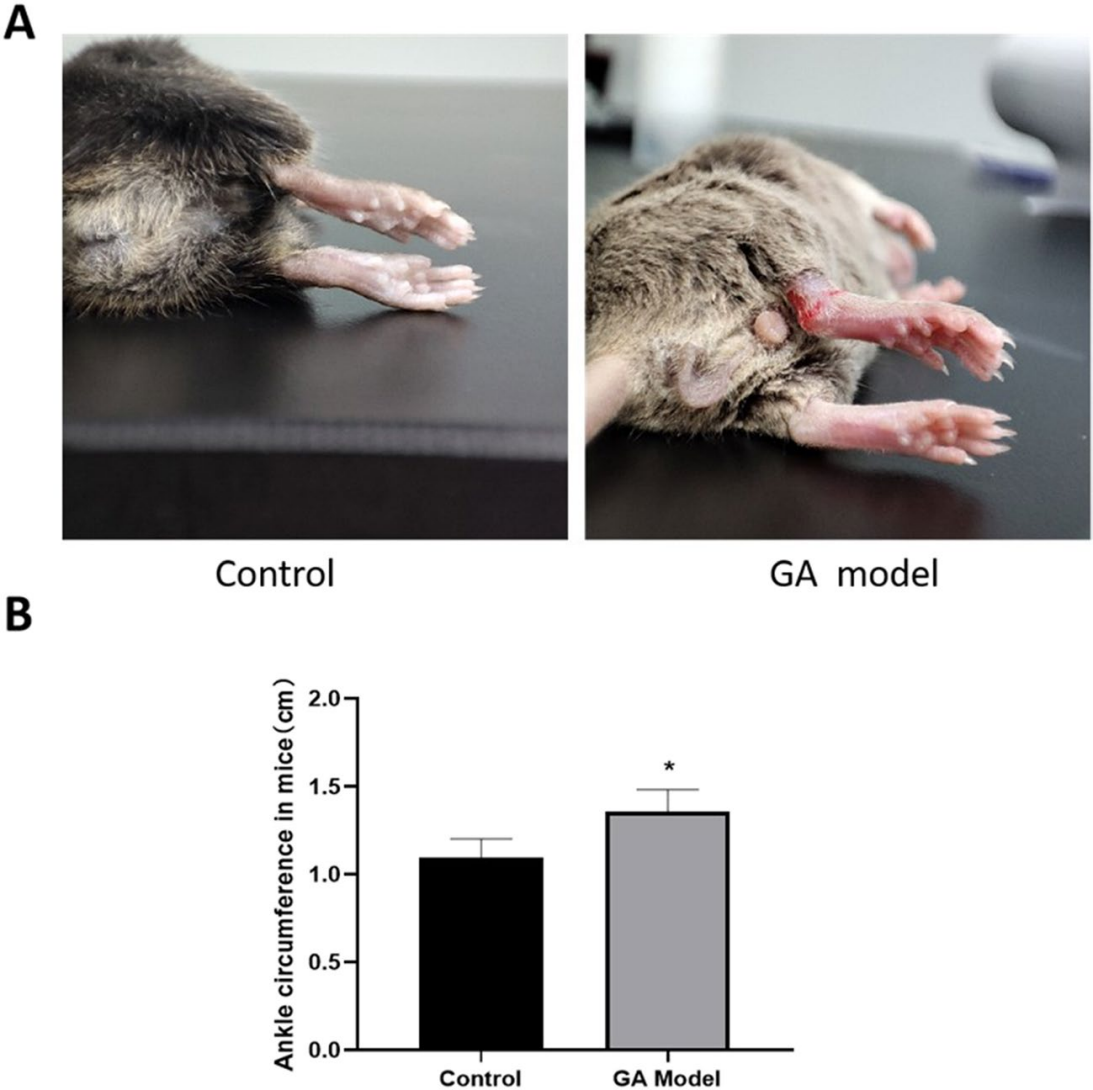
The successful establishment of GA model in mice. **A** The ankle joint picture of mice in the control and GA model groups. **B** The ankle circumference of the mice in the control and GA model groups was evaluated (*p < 0.05 vs Control)

GA model was constructed in mice by injecting 28 µL MSU crystals per animal into the ankle joint cavity at a concentration of 25 mg/mL. Before modeling, smooth hair, normal appetite, and nice mental status were observed in all mice, with no swelling of joints. After modeling, mice showed obvious redness and swelling in the ankle joint without any abnormality in hair color, appetite, or mental state (Fig. 6A). Moreover, compared to control, the ankle circumference of the mice after modeling was significantly increased (Fig. 6B).

### Stefin B overexpression alleviated the GA symptom in mice

**Fig. 7:**
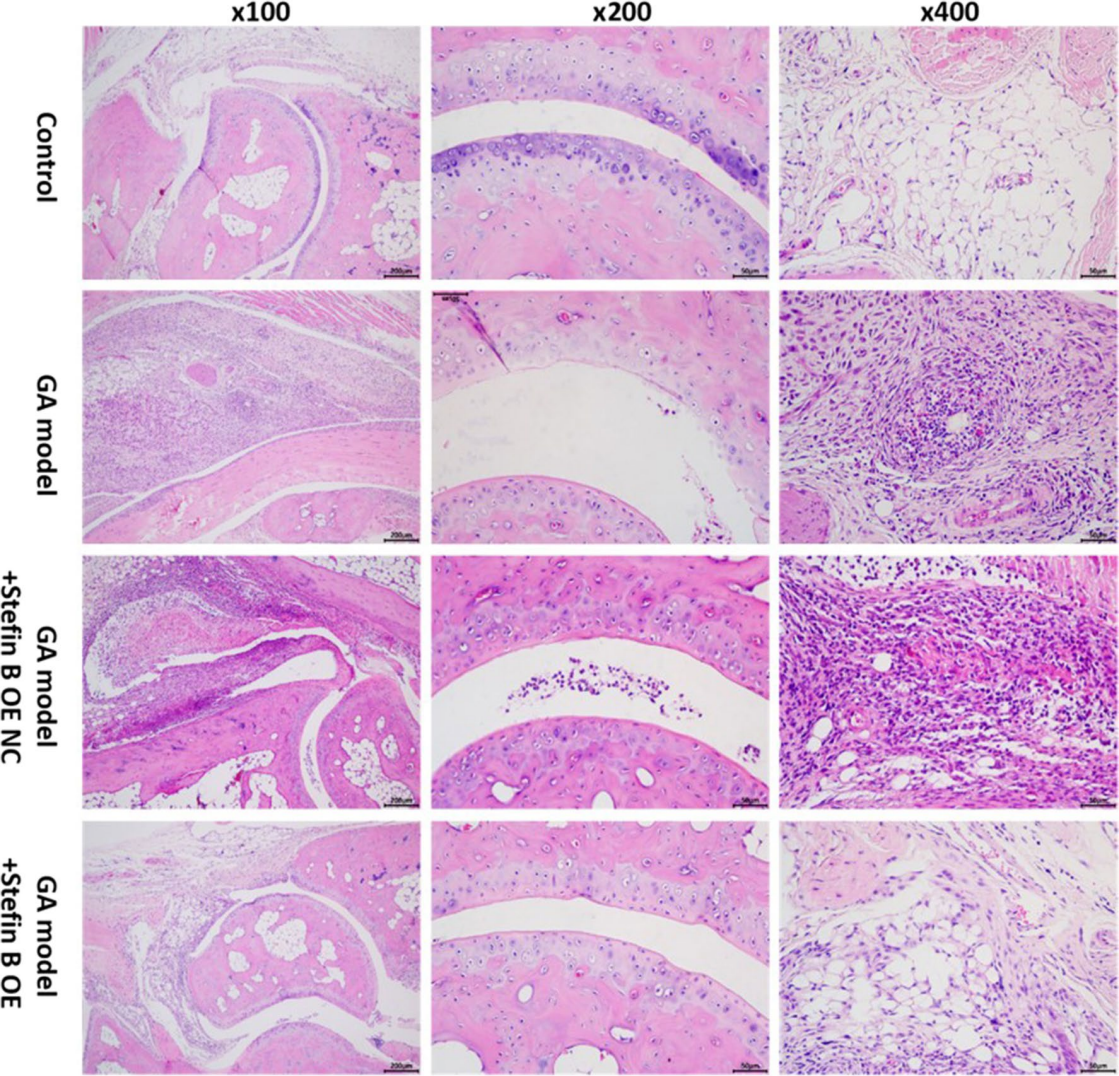
Stefin B overexpression alleviated the GA symptom in mice. The pathological state in the ankle joint tissue of mice was checked by HE staining assay

As shown in Fig. 7, in the GA model and GA model + Stefin B OE NC group, significant infiltration of inflammatory cells, swollen morphology of synovial cells, and ecchymosis blood vessels were observed, accompanied by large numbers of contents infiltrated into the joint cavity, enlarged cavity, and rough cartilage surface. In the control and GA model + Stefin B OE groups, no infiltration of inflammatory cells was observed, with rare swollen synovial cells, hardly any contents infiltrated into the joint cavity, and smooth cartilage surface. The GA symptom in mice was alleviated by the overexpression of Stefin B.

### Stefin B overexpression repressed the M1 polarization and facilitated the M2 polarization of macrophages in the ankle joint tissue of GA mice

**Fig. 8:**
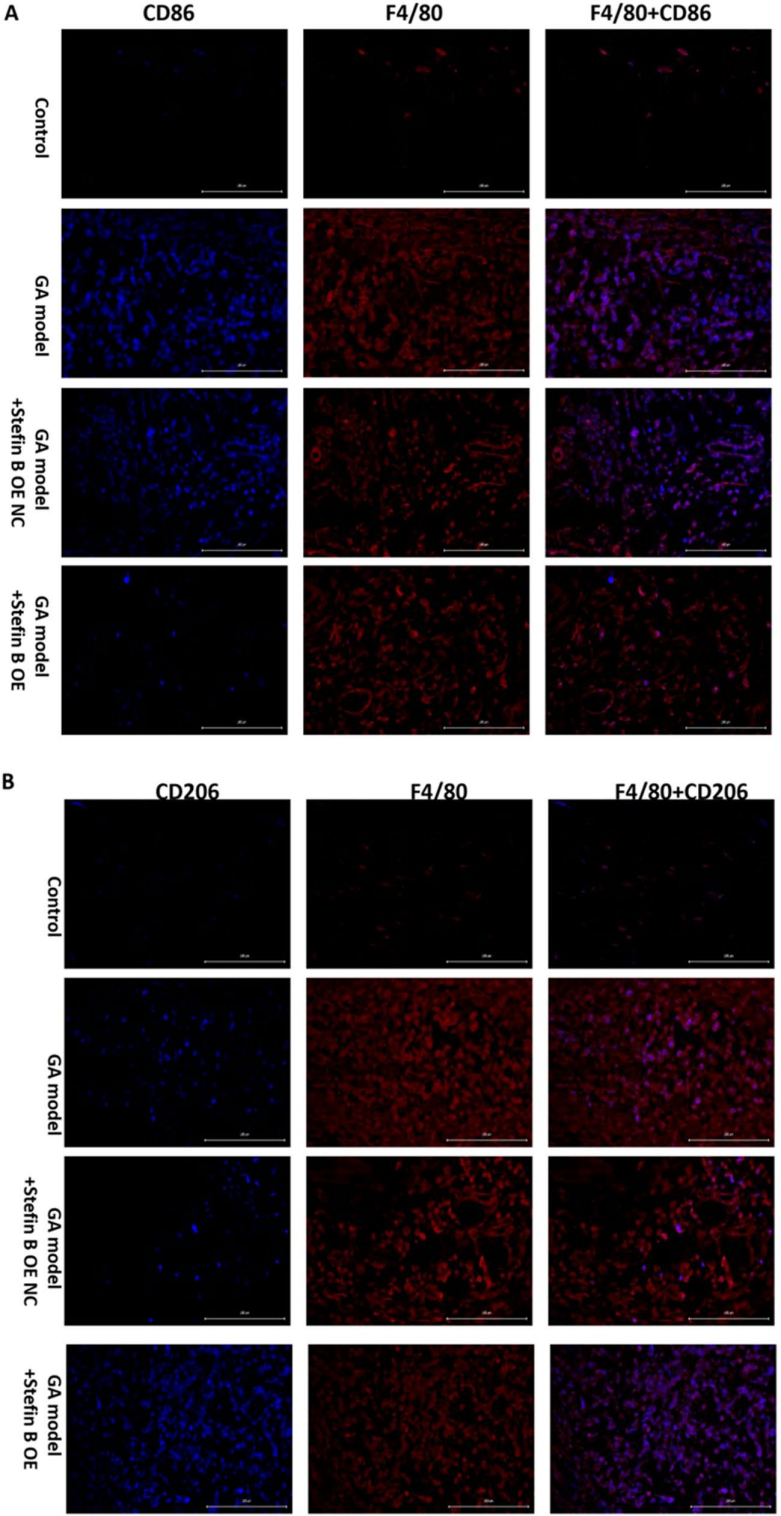
Stefin B overexpression repressed the M1 polarization and facilitated the M2 polarization of macrophages in the ankle joint tissue of GA mice. **A** The expression of CD86 and F4/80 in macrophages was detected by the double immunofluorescence assay. **B** The expression of CD206 and F4/80 in macrophages was detected by the double immunofluorescence assay

As shown in Fig. 8, the expression of CD206 or CD86 was represented by blue fluorescence, while the expression of F4/80 was represented by red fluorescence. Compared to control, CD86 was found dramatically upregulated in GA mice, the level of which was markedly repressed in the Stefin B OE group (Fig. 8A). Furthermore, compared to GA model, the level of CD206 was significantly increased in the Stefin B OE group (Fig. 8B). These data suggested that the M1 polarization was repressed, and the M2 polarization was facilitated in the ankle joint tissue of GA mice by the overexpression of Stefin B.

### Stefin B overexpression suppressed the inflammation in GA mice

**Fig. 9:**
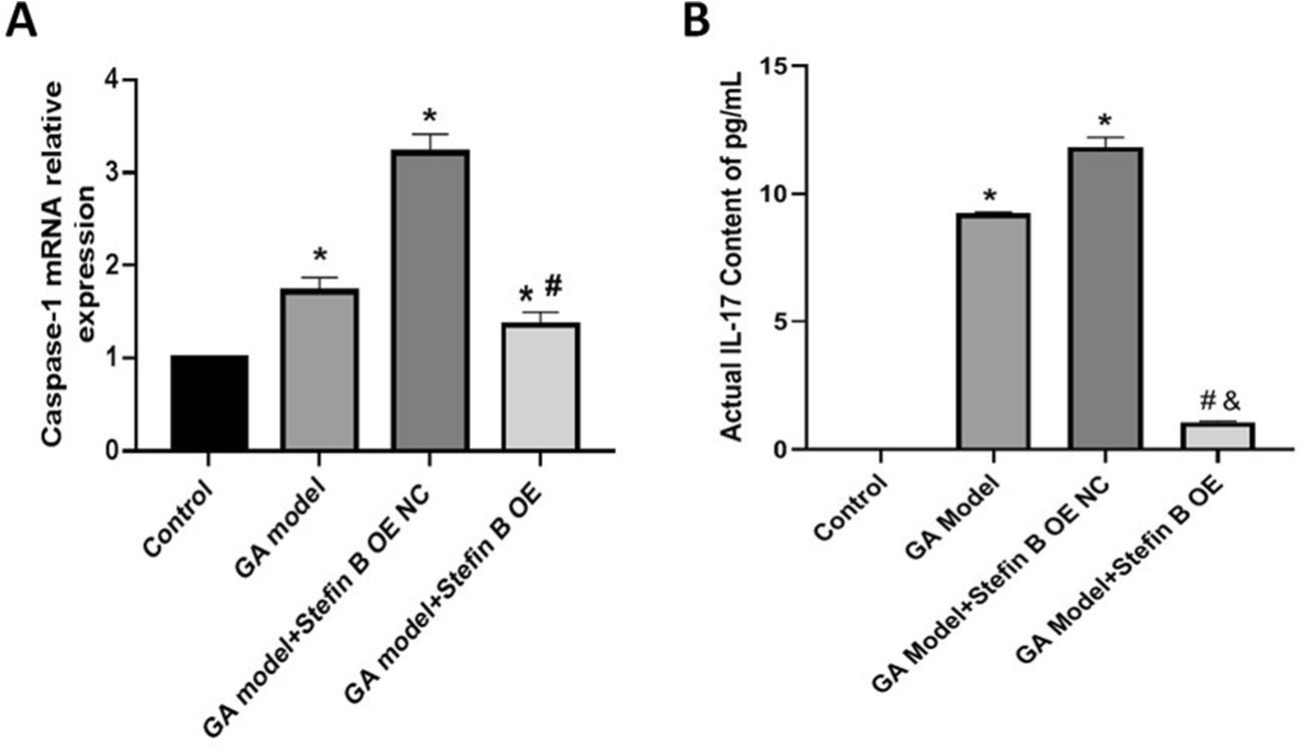
Stefin B overexpression suppressed the inflammation in GA mice. **A** The mRNA level in the ankle joint tissue was checked by RT-PCR assay. **B** The serum IL-17 level was detected by the ELISA assay (*p < 0.05 vs Control, #*p* < 0.05 vs GA model, &*p* < 0.05 vs GAmodel + Stefin B OE NC)

As shown in Fig. 9A, compared to control, the mRNA level of Caspase-1 was markedly increased in GA mice. Compared to the GA model and GA model + Stefin B OE NC group, the mRNA level of Caspase-1 was signally repressed in the GA model + Stefin B OE group. Furthermore, compared to control, the release of IL-17 was notably increased in GA mice. Compared to the GA model and GA model + Stefin B OE NC group, the release of IL-17 was significantly repressed in the GA model + Stefin B OE group.

## Discussion

Stefin B is an endogenous inhibitor of cysteine proteinases, and the development of diseases will be impacted by the competitive inhibition between Stefin B and cathepsin. It is recently reported that apoptosis, immunoregulation, and autolysis regulation of matrix metalloproteinases (MMPs) are important pathways for Stefin B to participate in the progression of diseases, among which immunoregulation has attracted great attention (Maher et al. 2014a). Macrophages are widely located on the mucosal surface of the coelom and are one of the important innate immune cells in humans. Macrophages are highly plastic with flexibility in polarization. In the present study, in order to verify the regulatory effect of Stefin B on macrophage polarization, M0, M1, and M2 macrophages were obtained from THP-1 cells by stimulation. It was found that Stefin B was poorly expressed in M1 macrophages and highly expressed in M2 type macrophages, which indicated that the expression of Stefin B was affected by the macrophage polarization.

In the present study, M0 macrophages were transfected with pcDNA3.1-Stefin B and siRNAs targeting Stefin B. CD206, a surface marker of M2 macrophages, was found significantly upregulated in M0 macrophages transfected with pcDNA3.1-Stefin B, indicating that M2 polarization was facilitated by Stefin B. The adaptive immune responses are activated by macrophages by releasing various cytokines, participating in inflammatory responses and maintaining homeostasis. The secretion of a large number of cytokines, such as TNF-α, is an important characteristic of M1 macrophages, while the release of anti-inflammatory molecules, such as IL-10, to promote tissue growth and inhibit inflammation is the characteristic of M2 macrophages (Binnemars-Postma et al. 2018; Chistiakov et al. 2018; Yunna et al. 2020). A previous study showed that in Stefin B-deficient bone marrow–derived macrophages (BMDMs), the induction of LPS-induced nitric oxide (NO), a kind of pro-inflammatory signal, was upregulated, with declined expression of IL-10 (Maher et al. 2014b). Moreover, phosphorylation of ERK and p38 MAPK was significantly reduced in Stefin B-deficient macrophages, as well as the phosphorylation of STAT-3. These findings suggest that Stefin B affects the expression of anti-inflammatory IL-10 under the action of LPS as the TLR4 agonist and exert an important role in suppressing inflammation. In order to further clarify the effect of expression of Stefin B on the inflammatory factors released by macrophages, we found that the secretion of IL-6 was facilitated in Stefin B-knockdown M0 macrophages, while the production of IL-10 was increased in Stefin B-overexpressed M0 macrophages, which indicated that Stefin B regulated the polarization of macrophages with a regulatory effect on inflammatory responses.

The onset of GA is triggered by MSU, which interacts with macrophages and releases a series of inflammatory factors, especially IL-1, to induce neutrophils to migrate to the synovial tissue (So and Martinon 2017). Recruited macrophages tend to be polarized to M1 macrophages to activate the NLRP3 inflammasome and induce the production of pro-inflammatory factors, such as IL-1β, TNF-α, CD68, and iNOS, leading to the deterioration of GA (Liu et al. 2017, 2019). Thus, reducing the inflammatory cytokines by inhibiting M1 polarization and suppressing the NLRP3 inflammasome is considered an ideal strategy for treating GA (Desai et al. 2017). Meanwhile, M2 polarization is triggered in macrophages by IL-4 and IL-10, which further release anti-inflammatory factors such as TGF-β1 (Ohya et al. 2022). Moreover, changes in M1/M2 phenotype are associated with the pathogenesis of GA, however, with mechanism of action unclaimed. In the present study, Stefin B was found to regulate the polarization of macrophages and play a certain inhibitory role in the inflammatory responses.

Stefin B is an intracellular inhibitor of cysteine cathepsin. Chu et al. found that in acute GA, the cathepsin B/cystatin C system is highly correlated with markers of joint inflammation, indicating the pathological roles of cathepsin B and cystatin C in inflammation (Chu et al. 2010). In order to reveal the therapeutic effect of Stefin B on GA, GA was established in mice by injecting MSU into the ankle joint cavity, followed by administering the adenovirus containing pcDNA3.1-Stefin B into the ankle joint. In the Control and the GA model + Stefin B OE group, no infiltration of inflammatory cells was observed, with rare swollen synovial cells and smooth cartilage surface, which suggested that the pathological damage of the ankle joint in mice was alleviated by Stefin B.

It is reported that in Stefin B-deficient mice, the early activation of microglia is prior to the loss of neurons, indicating that the inhibitor of Stefin B might play a role in the communication between microglia and neuron (Kopitar-Jerala 2015). Detailed analysis of Stefin B-deficient microglia activation revealed that the proportions of both pro-inflammatory M1 and anti-inflammatory M2 microglia were significantly increased in the brains of Stefin B-deficient mice. In the present study, Stefin B was found to alleviate GA by regulating the M1/M2 polarization of macrophages and affecting the release of inflammatory factors. In recent years, studies have confirmed that acute GA is closely related to the NLRP3/ASC/caspase-1 inflammasome axis (Reber et al. 2014; Yang et al. 2018; Zhou et al. 2018). In the present study, the mRNA level of caspase-1 in NLRP3 inflammasome was significantly downregulated in Stefin B-overexpressed GA mice, accompanied by a decreased serum IL-17 level, which is closely correlated to arthritis. Interestingly, compared to si-Stefin B NC, IL-1β level was markedly reduced in the si-Stefin B group. However, compared to Stefin B OE NC, IL-1β level was also markedly reduced in the Stefin B OE group. These results suggested that IL-1β level in macrophages was either suppressed or induced by Stefin B (Fig S1A). Furthermore, caspase-1 was markedly upregulated by Stefin B overexpression and markedly downregulated by Stefin B knockdown in macrophages (Fig S1B), suggesting that the suppressed mRNA level of caspase-1 in NLRP3 inflammasome observed in Stefin B-treated GA model might be induced by enhanced M2 polarization of macrophages, not directly by Stefin B. These data implied that Stefin B might reduce the release of arthritis-related inflammatory factor IL-17 and repressed the expression of caspase 1 by enhancing the M2 polarization of macrophages, thereby exerting the therapeutic effect on GA.

## Conclusion

Our data suggested that Stefin B alleviated the gouty arthritis in mice by inducing the M2 polarization of macrophages and inhibiting the NLRP3 inflammasome. The present study revealed the mechanism of Stefin B in the treatment of GA from the perspective of changes in macrophage phenotype.

### Supplementary Information

Below is the link to the electronic supplementary material.

Supplementary file1 (JPG 345 KB)

## Data Availability

Data will be available from the corresponding author on request.
