# Supplementary figures and images for: Stefin B alleviates the gouty arthritis in mice by inducing the M2 polarization of macrophages
Source: Naunyn Schmiedebergs Arch Pharmacol. 2024 Jan 31;397(8):5677–88. doi: 10.1007/s00210-023-02911-w (PMC11329408; doi:10.1007/s00210-023-02911-w)

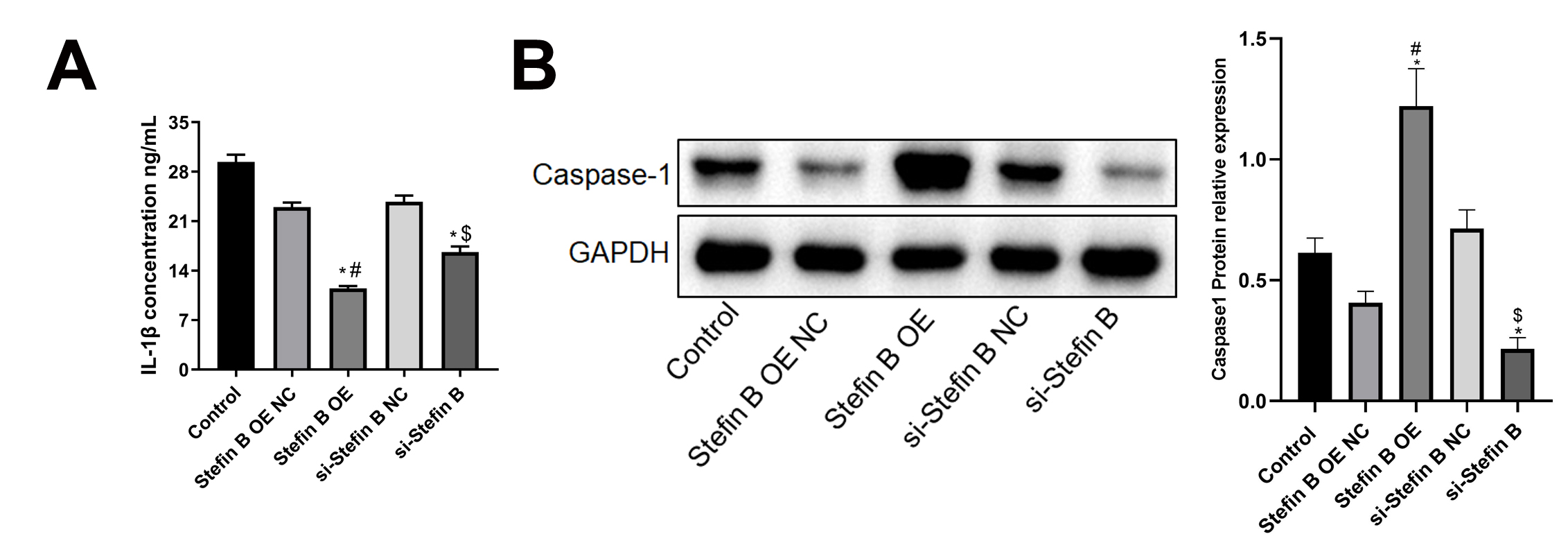

Supplement: Supplementary file 1 — Supplementary file1 (JPG 345 KB) [file 210_2023_2911_MOESM1_ESM.jpg]
